# Remarks on Peinado *et al.*'s Analysis of J3Gen

**DOI:** 10.3390/s150306217

**Published:** 2015-03-13

**Authors:** Joaquin Garcia-Alfaro, Jordi Herrera-Joancomartí, Joan Melià-Seguí

**Affiliations:** 1 Institut Mines-Telecom, Telecom SudParis, CNRS Samovar UMR 5157, 9 Rue Charles Fourier, 91000 Evry, France; 2 Department of Information and Communications Engineering, Universitat Autònoma de Barcelona, Edifici Q, Campus de Bellaterra, 08193 Bellaterra, Spain; E-Mail: jordi.herrera@uab.cat; 3 Internet Interdisciplinary Institute, Universitat Oberta de Catalunya, Roc Boronat 117, 08018 Barcelona, Spain; E-Mail: melia@uoc.edu

**Keywords:** network security, wireless security, cryptography

## Abstract

Peinado *et al.* analyzed the security of the J3Gen pseudorandom number generator proposed by Melià-Seguí *et al.*, and claimed weaknesses regarding its security properties. They also presented a deterministic attack based on the decimation of the J3Gen output sequences. We show that the assumptions made by Peinado *et al.* are not correct and that the proposed deterministic attack against J3Gen does not hold in practice.

## 1. Introduction

The Electronic Product Code Class 1 Generation 2 [1] (EPC Gen2 for short) is a passive low-cost radio-frequency identification (RFID) technology for automated identification over Ultra High Frequency (UHF) interfaces. EPC Gen2 compliant RFID tags are passive electronic labels powered by the electromagnetic field of RFID readers, with a typical reading distance of up to five meters. The main constraints to integrating security features on-board of EPC Gen2 tags are power consumption, performance and compatibility requirements, which can be summarized in the cost of the security features. EPC Gen2 tags only consider two main security elements: a 16-bit pseudorandom number generator and password-protected operations (using the pseudorandom sequences as a cipher tool). The pseudorandom sequences are also used as an anti-collision mechanism for inventorying processes and to acknowledge other EPC Gen2 specific operations. The on-board 16-bit pseudorandom number generator is, therefore, the crucial component that guarantees the security of a Gen2 tag.

In [2,3] Melià-Seguí *et al.* presented J3Gen, a pseudorandom number generator for low-cost passive RFID technologies. It is based on a linear feedback shift register (LFSR) configured with a multiple-polynomial tap architecture fed by a physical source of randomness, achieving a reduced computational complexity and low-power consumption as required by the EPC Gen2 standard. J3Gen is intended for security, improving the one-time-pad cipher unpredictability. The security of this generator was very recently claimed by Peinado *et al.* to be weak in [4,5]. In their work, Peinado *et al.* also present a deterministic attack based on the decimation of the J3Gen output sequences. We show in this letter that the claims by Peinado *et al.* do not hold and that their attack is not feasible in practice.

**Organization**—Section 2 outlines the design and main parameters of J3Gen. Section 3 summarizes the claims by Peinado *et al.* and provides our remarks. Section 4 closes the letter.

## 2. J3Gen

**Figure 1.**
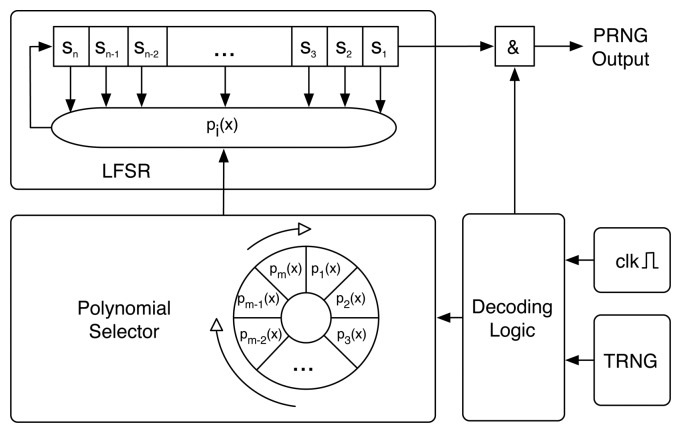
Block diagram of J3Gen.

J3Gen is a pseudorandom number generator design for low-cost passive RFID technologies. It is based on an *n*-cell linear feedback shift register (LFSR) configured with a multiple-polynomial tap architecture fed by a physical source of randomness. Figure 1 depicts a block diagram of the J3Gen design.

To thwart cryptanalysis, J3Gen handles the linearity of the LFSR module via the physical source of randomness (denoted as TRNG in Figure 1). The physical source of randomness generates *true random bits* (hereinafter denoted as *trn* bits). The *trn* bits control the change of polynomials, preventing the linear behavior of the LFSR. A set of *m* polynomials with degree *n* is assumed. Polynomials are selected following a wheel. When *trn* is 0, the polynomial selector rotates one position. When *trn* is 1, the polynomial selector rotates two positions. The rotations are performed every *ℓ* cycles, such that 1 ≤ *ℓ* < *n*. The security of J3Gen mainly depends on properly selecting the appropriate values of *n* (size of the LFSR), *m* (number of polynomials implemented by J3Gen) and *ℓ* (number of LFSR cycles prior the polynomial change).

## 3. Remarks on the Peinado *et al.* analysis

Peinado *et al.* claim in their analysis that in case that *ℓ* holds one, i.e., the feedback polynomial of J3Gen changes at every clock cycle, then “*the true random bit trn does no provide any randomness to the process, and each feedback polynomial will be periodically applied with period m*”. They also claim that when *ℓ* holds one, then *“the m feedback polynomials are applied consecutively, i.e., [p*_1_*(x)*, *p2(x),…, p_m_(x), p*_1_*(x)*, *p2(x),…]*”. The claims are wrong. As stated in [2,3], *“the internal J3Gen modules have different activation and deactivation timings*”, meaning that the clock signals controlling J3Gen (*i.e.*, the LFSR clock, as well as the clock of the physical source of randomness, and the one controlling the selection of polynomials) are not synchronized. Therefore, the list of polynomials (no matter the value of *ℓ*) would never be applied as suggested in [4,5]. This makes infeasible the deterministic attack proposed by Peinado *et al.*, as well as all the remainder figures, algorithms and boundaries provided in their analysis of J3Gen.

Furthermore, it is important to recall that the scenario envisioned by Peinado *et al.*, *i.e.*, scenario in which *ℓ* holds one, would mean that J3Gen generates true random bits at the same speed that it produces pseudorandom bits. This situation is not feasible in practice, since it is expected that the clock of the physical source of randomness is at a lower frequency, due to the energy constraints of EPC Gen2 RFID labels. Otherwise, the output of the source of true randomness would not need the assistance of J3Gen to produce *nonces*. The rationale for discarding the *ℓ* = 1 scenario is not driven by security purposes, but rather by the power constraints of the devices, as already stated in [2,3].

## 4. Conclusions

The Peinado *et al.* analysis of J3Gen in [4,5] is not correct. The analysis wrongly assumes that the clock signals guiding the main blocks of J3Gen, *i.e.*, the LFSR block, the physical source of randomness, and the Polynomial Selector are synchronized. As stated in [2,3], they are not. This misunderstanding is used by Peinado *et al.* to propose a deterministic attack based on the decimation of the J3Gen output sequences. Given that the main assumption to build the decimation attack is wrong, their deterministic approach against J3Gen is not feasible. Furthermore, the scenario assumed by their attack would require the generation of the same number of true random bits than pseudorandom bits. According to [3], the closer the value of *ℓ* to *n* (being *ℓ* the number of LFSR cycles prior the update of polynomials, and *n* the degree of the polynomials), the lower the security of J3Gen to face brute force attacks. The closer the value of *ℓ* to 1, the higher the security of J3Gen to face such attacks. The value *ℓ* = 1 is given in [3] as a boundary. If such a situation was possible, the design of J3Gen on EPC Gen2 environments would not even be necessary. J3Gen would simply output the true random sequences from the thermal-noise generator, validating the statement in [2,3] that the *ℓ* = 1 is the most secure option of J3Gen. All other suggestions in the security analysis by Peinado *et al.* were already provided in the original work (*cf.* references [2,3] and citations thereof).
